# Gain and Loss of Phototrophic Genes Revealed by Comparison of Two *Citromicrobium* Bacterial Genomes

**DOI:** 10.1371/journal.pone.0035790

**Published:** 2012-04-27

**Authors:** Qiang Zheng, Rui Zhang, Paul C. M. Fogg, J. Thomas Beatty, Yu Wang, Nianzhi Jiao

**Affiliations:** 1 State Key Laboratory of Marine Environmental Science, Xiamen University, Xiamen, People's Republic of China; 2 Department of Microbiology and Immunology, University of British Columbia, Vancouver, British Columbia, Canada; Duke-Nus Gradute Medical School, Singapore

## Abstract

*Proteobacteria* are thought to have diverged from a phototrophic ancestor, according to the scattered distribution of phototrophy throughout the proteobacterial clade, and so the occurrence of numerous closely related phototrophic and chemotrophic microorganisms may be the result of the loss of genes for phototrophy. A widespread form of bacterial phototrophy is based on the photochemical reaction center, encoded by *puf* and *puh* operons that typically are in a ‘photosynthesis gene cluster’ (abbreviated as the PGC) with pigment biosynthesis genes. Comparison of two closely related *Citromicrobial* genomes (98.1% sequence identity of complete 16S rRNA genes), *Citromicrobium* sp. JL354, which contains two copies of reaction center genes, and *Citromicrobium* strain JLT1363, which is chemotrophic, revealed evidence for the loss of phototrophic genes. However, evidence of horizontal gene transfer was found in these two bacterial genomes. An incomplete PGC (*pufLMC*-*puhCBA*) in strain JL354 was located within an integrating conjugative element, which indicates a potential mechanism for the horizontal transfer of genes for phototrophy.

## Introduction

Anoxygenic phototrophic bacteria were proposed to have emerged approximately 3 billion years ago and to be the ancestor of all photosynthetic organisms [1]–[3]. Aerobic anoxygenic phototrophic (AAP) bacteria, which probably evolved after the accumulation of oxygen in the earth's biosphere, are widely distributed in the euphotic zone of the ocean, and are thought to be significant players in marine carbon cycling [4]–[7]. To date, all AAP bacteria that have been discovered belong to the *Proteobacteria*, and the majority of cultured AAP strains are members of the *α-proteobacteria*. Genome sequencing has revealed that AAP bacteria, like purple photosynthetic bacteria, contain a highly conserved ∼40 to 50 kb "photosynthesis gene cluster" (PGC) [8]. The heart of anoxygenic phototrophy is the reaction center, encoded by the *puf* and *puh* operons. So far, there are two prominent viewpoints about the evolution of *Proteobacteria*: the first is that phototrophic ancestors lost phototrophy genes and thus became chemotrophic [8], [9]; the other is that chemotrophic bacteria acquired phototrophy genes via horizontal gene transfer (HGT) and therefore became phototrophic. For example, the *Rubrivivax gelatinosus* (*β-proteobacteria*) phototrophy genes were suggested to have originated in the photosynthetic *α-proteobacteria* [8], [10], [11]. Although these two viewpoints are not mutually exclusive, either one could explain the scattered distribution of phototrophy in phylogenetic trees.

**Figure 1:**
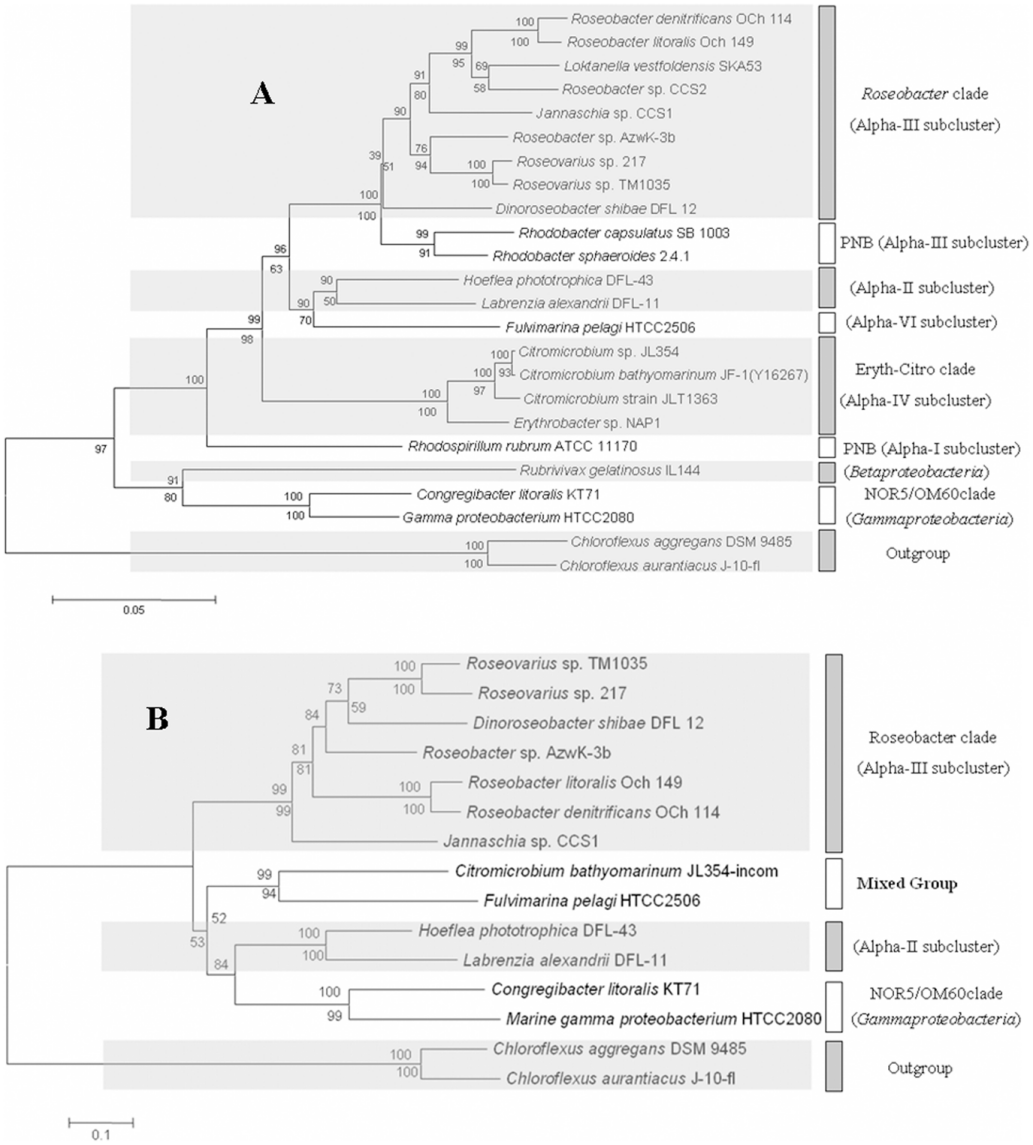
Neighbor joining phylogenetic analysis of 16S rRNA gene sequences from all whole genome-sequenced marine AAP bacteria. (A). Scale bar represents 5% nucleotide substitution percentage. Neighbor joining tree of *pufC* gene sequences from all whole genome-sequenced marine AAP bacterial (B). Scale bar represents 10% nucleotide substitution percentage. Bootstrap percentages (≥50%) from both neighbor joining (above) and maximum parsimony (below) are shown.

The genus *Citromicrobium* is in the Alpha IV AAP bacteria subcluster (**Fig. 1A**), and it contains only one species, *C. bathyomarinum*. The strain JL354, which was isolated from the upper ocean water of the South China Sea [12], shares 99.6% 16S rRNA sequence identity with the type strain *C. bathyomarinum* JF-1 that was isolated from deep-sea hydrothermal vent plume waters [13]. A previous study proposed the existence of two PGCs in the *Citromicrobium* sp. JL354 genome sequence, one complete and the other incomplete [12]. Subsequently, the *Citromicrobium* strain JLT1363 was isolated from the South China Sea, and was found to have 98.1% and 98.0% 16S rRNA gene sequence identity to *Citromicrobium* sp. JL354 and *C. bathyomarinum* JF-1, respectively (**Fig. 1A**). Surprisingly, there is no PGC in the genome of *Citromicrobium* strain JLT1363 [14].

Here, by comparing the genomes of the AAP bacterium *Citromicrobium* sp. JL354 and the closely-related chemotrophic bacterium *Citromicrobium* strain JLT1363, we provide evidence that JLT1363 evolved from an AAP bacterium by loss of phototrophy genes to become heterotrophic. In addition, we speculate that the incomplete PGC of *Citromicrobium* sp. JL354 was obtained by HGT.

## Results and Discussion

### Overview of Citromicrobium genomes

The genome of *Citromicrobium* strain JLT1363 is very similar to *Citromicrobium* sp. JL354, in terms of genome size (3,117,324 bps and 3,273,334 bps, respectively), GC content (64.9% and 65.0%, respectively), and gene composition (**Fig. S1**). However, strain JL354 is capable of phototrophic growth (phototrophy) on some carbon sources whereas strain JLT1363 is incapable of phototrophy. Comparison of the two genomes revealed that the major differences are the presence of a ∼37 kb PGC, and a Mu-like prophage (about 40 kb in length) and adjacent sequences in *Citromicrobium* sp. JL354, which are absent from the genome of JLT1363.

### Phenotypic and genetic diversity in the AAP bacteria

AAP bacteria have been thought to be closely related to the purple non-sulfur bacteria, as exemplified by the presence of AAP strains in the *Roseobacter* clade (Alpha III), the Alpha II subcluster, the *β-proteobacteria* (which includes the photosynthetic *Rubrivivax gelatinosus*), and the NOR5 clade (*γ-proteobacteria*) (**Fig. 1A**). There also are AAP bacteria that possess atypical characteristics akin to the purple non-sulfur bacteria; *e.g*., *Roseobacter denitrificans* OCh 114 and *Dinoroseobacter shibae* DFL 12 are able to grow under anaerobic conditions, and *Congregibacter litoralis* KT71 induces the expression of its photosynthetic apparatus under semi-aerobic conditions [15]–[17]. In addition, AAP bacteria in the *Roseobacter* and NOR5 clades utilize light harvesting pigments and carotenoid biosynthesis pathways similar to those used by the purple non-sulfur (photosynthetic) bacteria [8], [18], [19].

To date, only AAP bacteria in the Eryth-Citro clade are true aerobic phototrophs, containing unusual carotenoid biosynthetic pathways and pigments, compared to the other clades [18], [19]. Interestingly, members of the Eryth-Citro clade possess the shortest and simplest PGC structure of the known AAP [18], and the light harvesting complex 2 (LH2) genes are absent, perhaps indicating that the AAP in this clade are younger than their counterparts in other clades.

### Multiple potential mechanisms for HGT

Exchange of genetic information via HGT usually occurs by three major mechanisms: transformation, transduction or conjugation [20]. By these methods microorganisms may gain exogenous genes allowing adaptation to changing environmental pressures, for example antibiotic and heavy metal resistance, or to simply broaden the genetic diversity of the population [21]–[23]. HGT is thought to be a significant process in the evolution and plasticity of bacterial genomes [20], [22]. Compared to members of the *Roseobacter* clade, which have diverse metabolic capabilities supported by large genomes [24], [25], the oligotrophs of the genus *Citromicrobium* have small, streamlined genomes and no known plasmids, potentially making them less adaptable [26].

In order to compete and survive in natural environments, *Citromicrobium* species may benefit from alternative strategies to enhance their genomes by HGT. *Citromicrobium* species possess at least four potential mechanisms of HGT, which could result in adaptation to changing conditions: a gene transfer agent (GTA); an integrative conjugative element (ICE); a Mu-like prophage; and a type IV secretion system (T4SS). These mechanisms are described in the following text.

**Figure 2:**
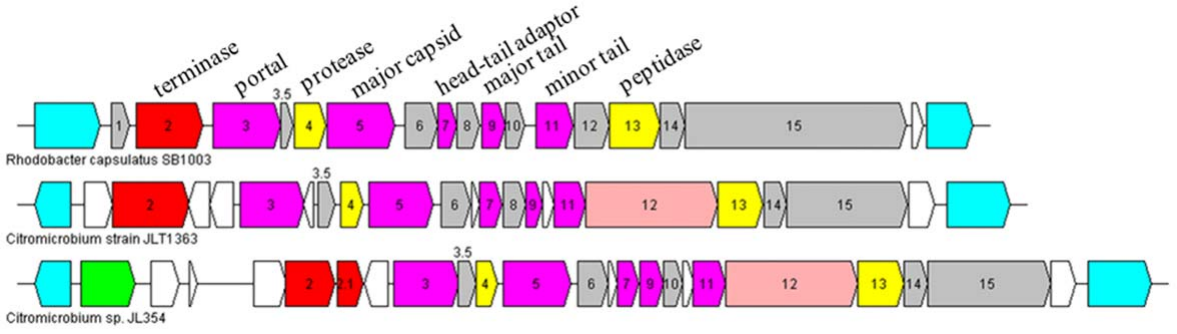
Organization of GTA structural genes in *R. capsulatus* and two *Citromicrobium* genomes (Acc No., JLT1363: NZ_AEUE01000002; JL354: NZ_ADAE01000023). Cyan, conserved genes upstream and downstream of the GTA structural gene cluster in *Rhodobacter* and Citro-Eryth clades; green, a putative transposase; red, terminase; purple, structural genes; yellow, protease and peptidase; white, hypothetical genes; gray, conserved hypothetical genes belonging to GTA.

GTAs are small, phage-like particles that transfer random, short stretches of genomic DNA (typically, 4 to 14 kb in length) between closely related bacteria, and which were first discovered in *Rhodobacter capsulatus* [27], [28]. Control of GTA-mediated genetic exchange is intimately intertwined with key host cell regulatory systems and GTA-like gene clusters are conserved in diverse genera, suggesting that they play an important role in evolution or adaptation of the bacterium [29]. Analysis of *Citromicrobial* genomes showed that there are complete GTA gene clusters present in both of the genomes examined here (**Fig. 2**). Overall, the structure of these clusters is very similar to that of the archetypal GTA in *R. capsulatus* (RcGTA) (**Fig. 2**).

**Figure 3:**
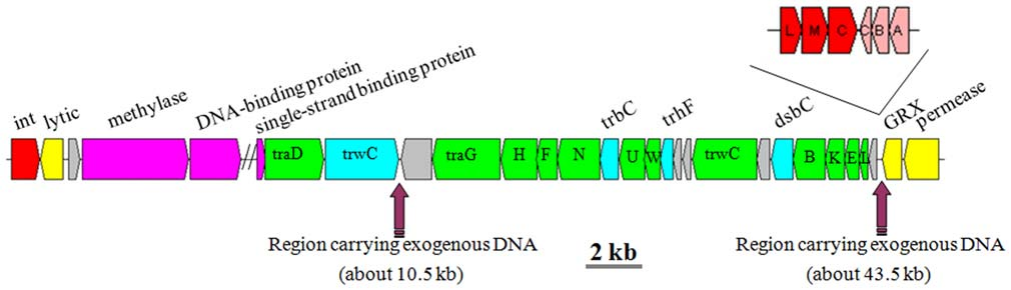
Genomic organization of core genes of a putative ICE found in the genome of *Citomcirobium* strain JLT1363. The two regions suggested to carry exogenous DNA are predicted to be involved in phage repression (10.5 kb) and heavy metal transport (43.5 kb) (Acc No.: NZ_AEUE01000004). The ICE in JL354 is in different contigs, but all the core genes after and including ‘single-strand binding protein’ are present (Acc No.: NZ_ADAE01000017). The incomplete PGC (*pufLMC-puhCBA*) only found in ICE of strain JL354. Red, yellow and pink: coding for phage-related genes; green and cyan: coding for plasmid-related genes.

Integrative and conjugative elements (ICEs) are self-transmissible mobile genetic elements that mediate HGT among prokaryotes, driving macro-evolutionary events [30], [31]. ICEs possess characteristics of both conjugative plasmids and temperate bacteriophages-ICEs transfer via conjugation like plasmids, but they cannot replicate autonomously and so integrate into the host chromosome like temperate bacteriophages [31]–[33]. Furthermore, ICEs have been reported to contain five intergenic hotspots where exogenous genes can be held [34]. Both of the *Citromicrobium* genomes examined here contain ICE-like gene clusters, and within the ICE of strain JLT1363 there are two putative hotspot regions One region (predicted to be involved in phage repression, NZ_AEUE01000004: (339285-349725)) is located between the *trwC* and *traG* genes, the other (heavy metal transport, NZ_AEUE01000004: (367759-410917)) is located adjacent to the *traBKEL* gene cluster (**Fig. 3**).

An inducible Mu-like prophage was identified in *Citromicrobium* sp. JL354 (NZ_ADAE01000018: complement (530353–567404), unpublished data). The complete prophage sequence is ∼37 kb in length with a GC content of 66.1%, and comprises of 57 predicted ORFs. Characterized Mu-like phages package host DNA from the regions immediately flanking the integrated prophage during prior to lytic release [35], [36]. The almost random replicative transposition characteristic of Mu-like phages results in the packaging of heterologous host DNA segments, which could conceivably play a role in the exchange of genetic information between hosts [35].

The *virB*-encoded T4SSs mediate interbacterial conjugative DNA transfer, and secretion of virulence factors into target cells [37]–[40]. The complete *virB* operon (*virB1-virB11*) and the *virD4* gene are required to form the macromolecular transfer apparatus and for a strain to serve as a donor [41], [42], but only a subset are required for recipient activity [43]. Seven genes (*virB2B3B4–virB6–virB9B10B11*) were found in the two *Citromicrobial* genomes studied, and these incomplete *virB* operons may play a role in importing DNA in *Citromicrobium*.

### Loss of phototrophy genes

The AAP may be considered as a transitional group between photosynthetic and chemotrophic bacteria, because phototrophy supports no more than 20% of cellular energy requirements under illuminated aerobic conditions [7]. The AAP bacteria mainly utilize organic matter for growth. In oligotrophic oceanic regions, AAP bacteria may have some advantages over chemotrophs, but in a eutrophic oceanic area or rich organic medium, they do not express phototrophy genes [44], and so prolonged growth in a eutrophic environment could allow for loss of phototrophy genes.

**Figure 4:**
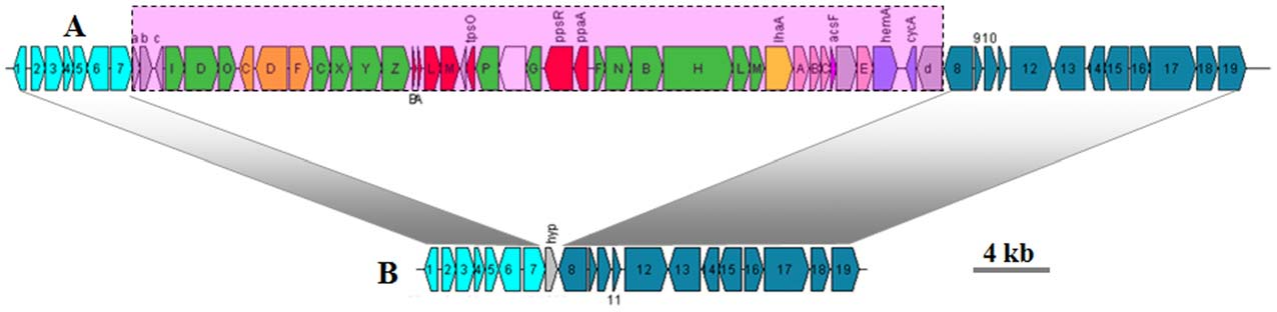
The difference between the PGC chromosomal region in *Citromicrobium* sp. JL354 (Acc No.: NZ_ADAE01000008) and the corresponding region in *Citromicrobium* strain JLT1363 (Acc No.: NZ_AEUE01000008). A , *Citromicrobium* sp. JL354; **B**, *Citromicrobium* strain JLT1363. As described in the text, this difference is attributed to the loss of the PGC in *Citromicrobium* strain JLT1363. Key to general classes of gene annotations: Green, *bch* genes; red, *puf* and regulator genes; pink, *puh* genes; orange, *crt* genes; blue, *hem* and *cyc* genes; yellow, *lhaA* gene; blank, uncertain or unrelated genes; grey, hypothetical protein. The horizontal arrows represent putative transcripts. Key to specific gene annotations: 1, zinc finger/thioredoxin putative; 2, cell division ATP-binding protein FtsE; 3, cell division transport system permease protein; 4, hypothetical protein ELI_11430; 5, 1-acyl-sn-glycerol-3-phosphate acyltransferase; 6, histidinol-phosphate aminotransferase; 7, homoserine O-acetyltransferase; 8, succinate-semialdehyde dehydrogenase (NAD(P)+); 9, hypothetical protein ED21_17902; 10, glutathione S-transferase family protein; 11, protein-methionine-S-oxide reductase; 12, membrane carboxypeptidase; 13, trypsin-like serine protease; 14, HflC protein; 15, integral membrane proteinase; 16, ATPase; 17, aldehyde oxidase and xanthine dehydrogenase molybdopterin binding; 18, ferrochelatase; 19, cytochrome P450. A, acetyltransferase, GNAT family protein; B, methionine biosynthesis protein MetW; C, glyoxalase/bleomycin resistance protein/dioxygenase; D, membrane anchored protein.

The complete PGC in the *Citromicrobium* sp. JL354 genome consists of two conserved subclusters, *crtCDF*-*bchCXYZ*-*pufBALM* and *bchFNBHLM*-*lhaA*-*puhABC* (**Fig. 4**), flanked by putative genes involved in cell division, transmembrane transport and signal transduction. Interestingly, in the JLT1363 genome we found exactly the same flanking gene organization but with a single hypothetical gene in place of the PGC (**Fig. 4**). It appears that either HGT (*i.e.*, JL354 obtained the PGC) or gene cluster loss (*i.e*., JLT1363 lost the PGC) occurred in *Citromicrobium*. To address these possibilities, phylogenetic analyses of strain JL354 PGC and 16S rRNA genes were carried out, and the trees were evaluated for congruency. The general topology of these phylogenetic trees was similar (**Fig. 1A** **, Fig. S2**), indicating co-evolution of the PGC with the 16S rRNA gene. These results indicate that *Citromicrobium* sp. JL354 acquired the complete PGC long before 16S rRNA phylogenetic divergence, and that relatively recent HGT does not account for the complete PGC in strain JL354. Therefore, the data suggest that *Citromicrobium* strain JLT1363 lost its PGC after divergence from a common ancestor shared with strain JL354.

HGT could not only increase the host genetic information but could also trigger chromosome recombination resulting in gene loss or duplication. For example, Mu-like phage lysogen formation involves transposition into almost random locations within the host genome, and if this were to occur within a gene or operon it would be likely to cause complete or partial loss of function at the insertion site [45]. Furthermore, transposing phages and other insertion sequences are known to be key factors in the reorganization and evolution of bacterial genomes [46]–[48]. The disruption of a single bacteriochlorophyll biosynthesis or reaction center gene in the PGC would lead to loss of phototrophy, and thus there are many potential sites for gene disruption that would lead to the absence of a selective advantage for the PGC as a whole. Subsequently, a non-functional PGC would be an energy drain during genome replication, conferring a selective advantage on progeny for the loss of this non-functional PGC in an environment allowing chemotrophic growth. Although further investigation is needed to elucidate the exact mechanism of this proposed PGC loss, our study could provide an explanation for the scattered evolutionary lineage for the presence and absence of phototrophy throughout the *Proteobacteria* clade, and may also explain the numerous closely-related phototrophic and chemotrophic strains [8].

### Gain of phototrophy genes

Coexistence of two different PGCs in one phototrophic bacterium has been found only in *Citromicrobium* sp. JL354 [12]. The complete PGC is typical, whereas the incomplete PGC consists of just the *pufLMC* and *puhABC* genes, which are contiguous (**Fig. 3**). However, in most other PGCs, including the complete PGC of the same strain, *pufLM* and *puhABC* are separated [18], and *pufC* is not present in Alpha IV AAP containing complete PCGs (*e.g.*, *Citromicrobium* sp. JL354 and *Erythrobacter* sp. NAP1). Furthermore the GC content of the *pufLM* and *puhABC* sequences in the incomplete PGC (0.62 and 0.66, respectively) is lower than that of the complete PGC (0.63 and 0.69, respectively). The nucleotide identity is less than 80% for all shared genes between the two PGCs, and the difference is higher than the genera level (15%) [49]. On the basis of the foregoing observations, we speculate that the incomplete PGC in *Citromicrobium* sp. JL354 was obtained via HGT. This hypothesis is strengthened substantially by further genomic analysis which revealed that the incomplete PGC in *Citromicrobium* sp. JL354 is located within one of the putative ICE intergenic hotspots **(** **Fig. 3** **)**. Further phylogenetic analysis showed that the *pufC* gene sequence belonging to the incomplete PGC is closest to *Fulvimarina pelagi* HTCC2506 (**Fig. 1B**), which indicates that the incomplete PGC genes may have been acquired from a *Fulvimarina-*related species (**Table 1**).

**Table 1:** Highest-scoring BLAST results (PSI-BLAST) using genes from the JL354 incomplete PGC (*pufLMC-puhABC*).

Incomplete PGC gene	Closest mach (AA identity)	AA identity with *F. pelagi* HTCC2506
*pufL*	Gamma proteobacterium NOR51-B 211/275 (77%)	203/271 (75%)
*pufM*	*F. pelagi* HTCC2506 226/319 (71%)	226/319 (71%)
*pufC*	*F. pelagi* HTCC2506 156/328 (48%)	156/328 (48%)
*puhA*	*F. pelagi* HTCC2506 100/250 (40%)	100/250 (40%)
*puhB*	*H. phototrophica* DFL-43 72/193 (38%)	71/198 (36%)
*puhC*	*Rps. palustris* DX-1 42/144 (30%)	41/144 (29%)

It has previously been suggested that purple photosynthetic bacteria were ancestral to the AAP [4]. Here, by comparison of two *Citromicrobial* genomes, we have shed light on how an AAP species may have lost phototrophic competence to become chemotrophic. In addition, we have also identified multiple potential HGT mechanisms and provided evidence for gain as well as loss of phototrophy genes. Therefore, it appears that influence of HGT on the presence of phototrophy genes has contributed to the genotypes and phenotypes of extant species of *Proteobacteria*. With the increasing scope and depth of genome and metagenome databases, future detailed analysis should further clarify the evolutionary history of phototrophy.

### Materials and Methods

All the genome information was obtained from the GenBank genome database. The GenBank accession numbers are: *Citromicrobium* sp. JL354 (ADAE00000000), *Citromicrobiom* strain JL1363 (AEUE01000000), *Loktanella vestfoldensis* SKA53 (NZ_AAMS00000000), *Dinoroseobacter shibae* DFL 12 (NC_009952), *Roseobacter denitrificans* OCh 114 (NC_008209), *Jannaschia* sp. CCS1 (NC_007802), *Roseobacter litoralis* Och 149 (NZ_ABIG00000000), *Roseovarius* sp. 217 (NZ_AAMV00000000), *Roseobacter* sp. CCS2 (NZ_AAYB00000000), *Roseobacter* sp. AzwK-3b (NZ_ABCR00000000), *Erythrobacter* sp. NAP1 (NZ_AAMW00000000), *Congregibacter litoralis* KT71 (NZ_AAOA00000000), Marine *gammaproteobacterium* HTCC2080 (NZ_AAVV00000000), *Rhodobacter sphaeroides* strain 2.4.1 (NC_007493), *Rhodobacter capsulatus* SB 1003 (NC_014034), *Chloroflexus aggregans* DSM 9485 (NC_011831), and *Chloroflexus aurantiacus* J-10-fl (NC_010175).

All the sequences collected from NCBI database were aligned using Clustal X and phylogenetic trees were constructed using the neighbour-joining and maximum-parsimony algorithms of MEGA software 3.0 [50]. The phylogenetic trees were supported by bootstrap for resampling test with 1000 replicates.

## Supporting Information

Figure S1 **Pie chart of protein categorization of predicted coding sequences in the strains JL354 (outer) and JLT1363 (inner) genomes respectively.**
(PPT)

Figure S2 **Maximum parsimony phylogenetic analysis of 27 core proteins (∼10 kb aa) in PGCs from GenBank database.** The core proteins are BchBCDFGHILMNOPXYZ-CrtCF-PufBALM-LhaA-PuhABCE-AscF. Bootstrap percentages from both maximum parsimony (above) and neighbor joining (below) are shown. Bar, 0.1 substitutions per amino acids position.
(PPT)
