# Supplementary figures and images for: Gain and Loss of Phototrophic Genes Revealed by Comparison of Two Citromicrobium Bacterial Genomes
Source: PLoS One. 2012 Apr 27;7(4):e35790. doi: 10.1371/journal.pone.0035790 (PMC3338782; doi:10.1371/journal.pone.0035790)

## Slide 1
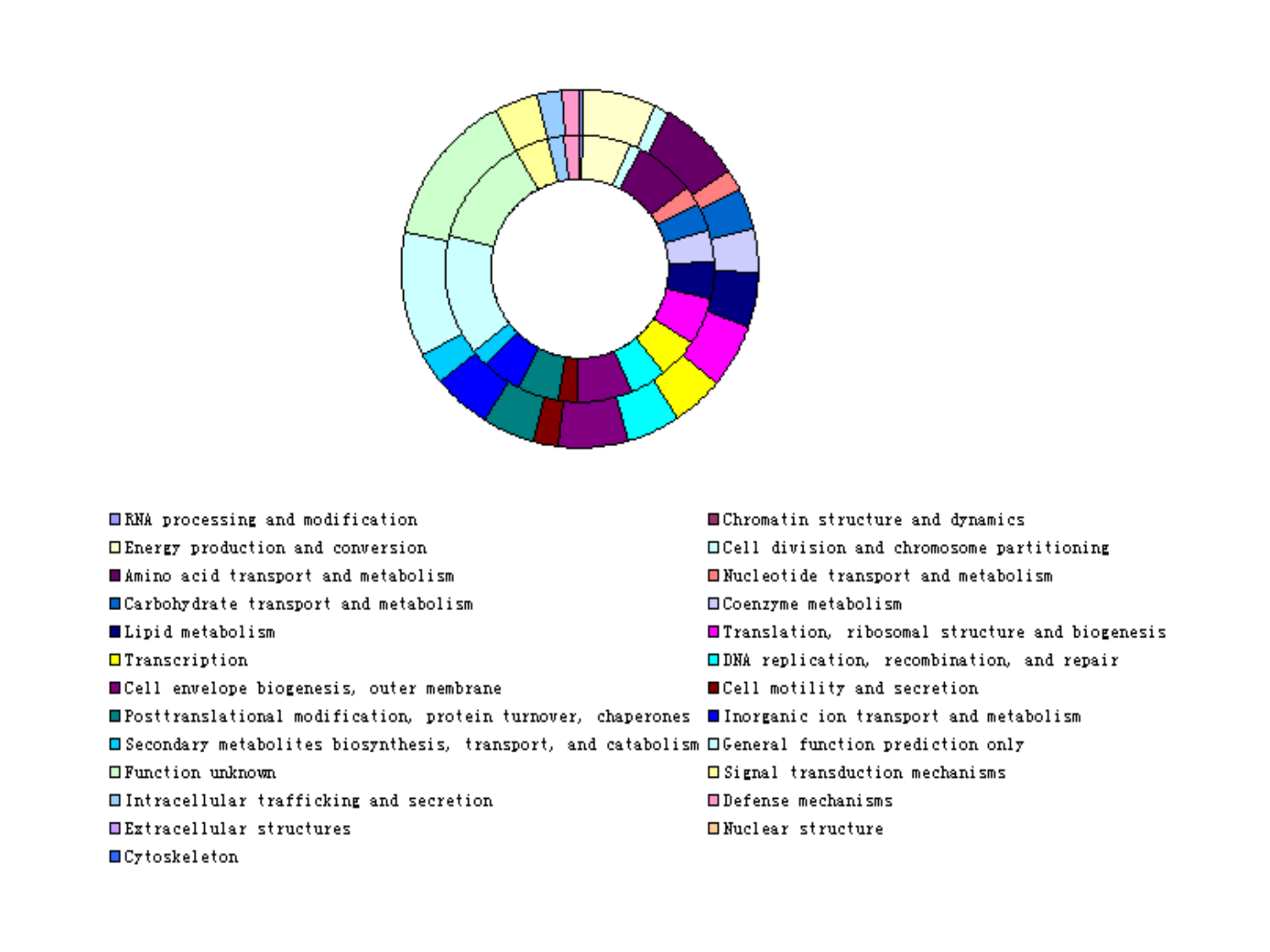

Supplement: Figure S1 — Pie chart of protein categorization of predicted coding sequences in the strains JL354 (outer) and JLT1363 (inner) genomes respectively. (PPT) [file pone.0035790.s001.ppt]

## Slide 1
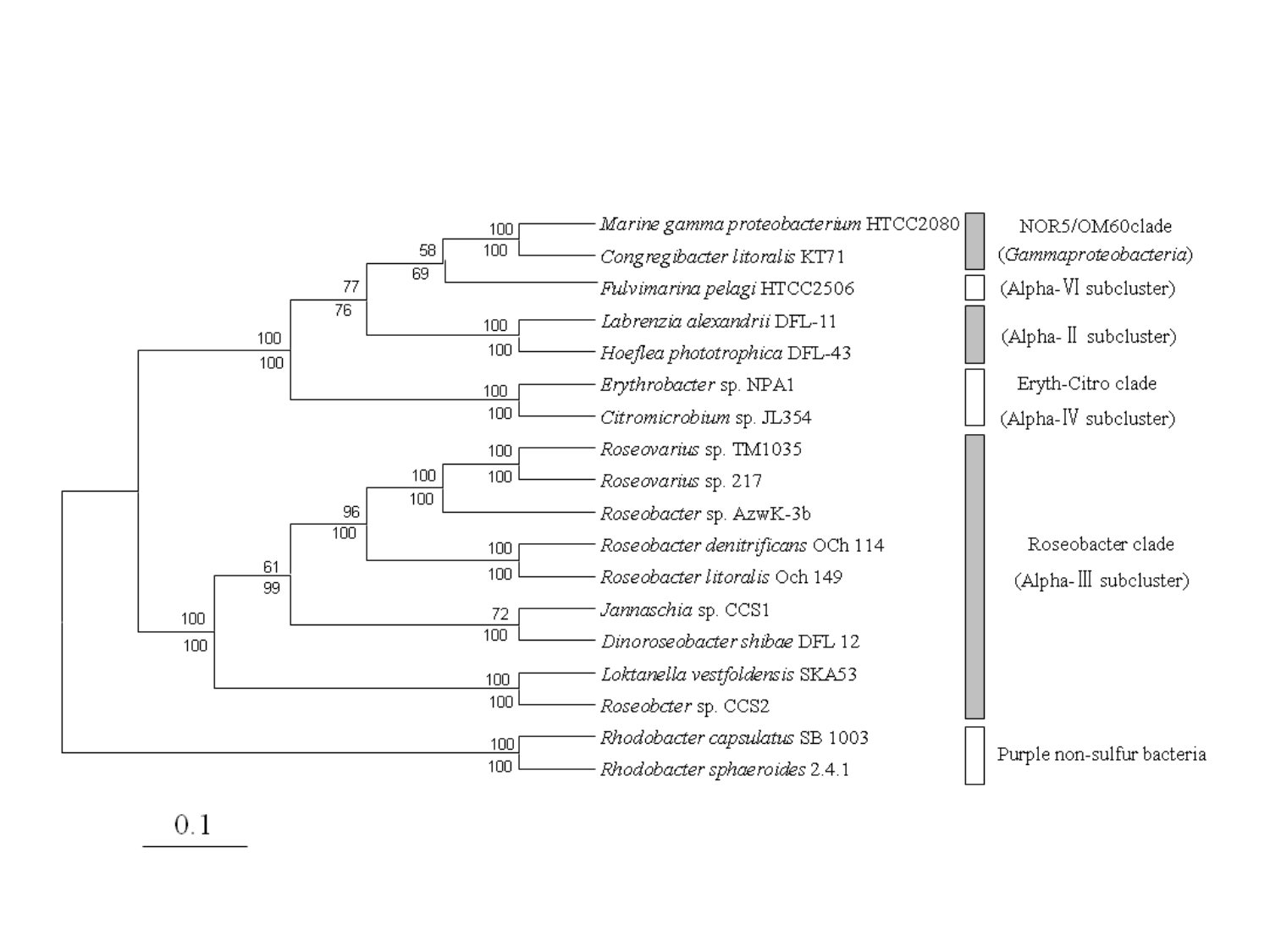

Supplement: Figure S2 — Maximum parsimony phylogenetic analysis of 27 core proteins (∼10 kb aa) in PGCs from GenBank database. The core proteins are BchBCDFGHILMNOPXYZ-CrtCF-PufBALM-LhaA-PuhABCE-AscF. Bootstrap percentages from both maximum parsimony (above) and neighbor joining (below) are shown. Bar, 0.1 substitutions per amino acids position. (PPT) [file pone.0035790.s002.ppt]
